# Integrating Diagnostic Approaches in Infant Bacterial Meningitis Caused by a Non-K1 *Escherichia coli*: A Case Report

**DOI:** 10.3390/antibiotics13121144

**Published:** 2024-11-28

**Authors:** Gianluca Vrenna, Marilena Agosta, Valeria Fox, Martina Rossitto, Venere Cortazzo, Serena Raimondi, Barbara Lucignano, Manuela Onori, Livia Mancinelli, Maria del Carmen Pereyra Boza, Vanessa Fini, Annarita Granaglia, Laura Lancella, Francesca Ippolita Calo’ Carducci, Costanza Tripiciano, Alberto Villani, Paola Bernaschi, Carlo Federico Perno

**Affiliations:** 1 Multimodal Laboratory Medicine, Bambino Gesù Children’s Hospital, IRCCS, 00165 Rome, Italy; gianluca.vrenna@opbg.net (G.V.); martina.rossitto@opbg.net (M.R.); 2 Microbiology and Diagnostic Immunology Unit, Bambino Gesù Children’s Hospital, IRCCS, 00165 Rome, Italy; marilena.agosta@opbg.net (M.A.); venere.cortazzo@opbg.net (V.C.); serena.raimondi@opbg.net (S.R.); barbara.lucignano@opbg.net (B.L.); manuela.onori@opbg.net (M.O.); livia.mancinelli@opbg.net (L.M.); mdcarmen.pereyraboza@opbg.net (M.d.C.P.B.); vanessa.fini@opbg.net (V.F.); annarita.granaglia@opbg.net (A.G.); paola.bernaschi@opbg.net (P.B.); carlofederico.perno@opbg.net (C.F.P.); 3 Infectious Disease Unit, Bambino Gesù Children’s Hospital, IRCCS, 00165 Rome, Italy; laura.lancella@opbg.net (L.L.); fippolita.calo@opbg.net (F.I.C.C.); costanza.tripiciano@opbg.net (C.T.); 4 General Pediatric and Infectious Disease Unit, Pediatric Emergency Medicine, Bambino Gesù Children’s Hospital, IRCCS, 00165 Rome, Italy; alberto.villani@opbg.net

**Keywords:** infant meningitis, non-K1 *Escherichia coli*, whole-genome sequencing, FilmArray

## Abstract

Background: Infant meningitis, particularly caused by *Escherichia coli*, remains a life-threatening condition, especially in premature and low-weight infants. Infections of the central nervous system can be fatal, necessitating prompt diagnosis and appropriate treatment. Acute infections caused by various pathogens, including *E. coli*, often present with similar clinical symptoms. The rapid identification of pathogens and their antimicrobial resistance mechanisms is critical for timely and effective treatment. We report the case of an 8-month-old patient who presented with fever, diarrhea, and convulsive seizures and was subsequently diagnosed with meningitis. Despite initial empirical treatment with ceftriaxone, the patient’s condition worsened. Methods: At Bambino Gesù Children’s Hospital, molecular diagnostic tools, including the FilmArray Meningitis/Encephalitis and Blood Culture Identification panels, were employed. Results: Although the Meningitis panel did not detect any pathogens due to the lack of the specific bacterial target, the off-label use of the Blood Culture Identification panel identified a non-K1 *Escherichia coli* strain carrying the CTX-M resistance gene, an extended-spectrum beta-lactamase (ESBL). Despite the rapid diagnostic approach and adjustment of antibiotic therapy, the patient succumbed to the infection due to the strain’s high virulence and multidrug resistance. Whole-genome sequencing further characterized the strain, revealing that it belonged to the ST131 group, a highly resistant and virulent strain associated with sepsis. Conclusions: This case highlights the importance of integrating advanced molecular diagnostics, such as whole-genome sequencing, with traditional methods to improve pathogen detection, especially in cases of emerging resistant strains that are not covered by standard diagnostic panels. It also emphasizes the need for the continuous adaptation of diagnostic tools to include non-K1 *E. coli* strains for more comprehensive and timely meningitis diagnosis.

## 1. Introduction

Neonatal meningitis is one of the most severe *Escherichia coli* infections, resulting in a mortality and morbidity rate of up to 25% [1], especially in premature and low-weight infants [2]. The *E. coli* strains capable of causing severe infections like meningitis usually express the K1 capsular antigen [3]. Most *E. coli* strains belong to sequence type complex 95 (STc) and to phylogroup B2. Within this STc, the presence of the K1 capsular antigen is the most important virulence factor [4]. We present a case of infant meningitis caused by non-K1 *E. coli*, in which the patient arrived in the emergency department’s red zone with convulsive seizures and in critical condition, providing insights into the identification, management, and prognosis of this severe bacterial infection. Accurate and rapid detection of the potential microorganism and its mechanisms of antimicrobial resistance is essential, particularly during suspected sepsis and meningitis. This allows for the prompt administration of appropriate therapy aimed at rapidly resolving the infection. The FilmArray Meningitis/Encephalitis Panel BioFire (bioMérieux) is a valuable tool for diagnosing meningitis caused by various pathogens, including *E. coli* (only the K1 strain, the most important), and is the only panel certified for cerebrospinal fluid (CSF). This syndromic panel is an easy-to-use multiplex PCR system designed for rapid testing, providing diagnostic results in a critically short period of time (1 h) from a small sample (0.2 mL). This panel tests cerebrospinal fluid (CSF) for 14 of the most common pathogens associated with community-acquired meningitis or encephalitis. In particular, it detects six bacteria (*Escherichia coli* K1, *Haemophilus influenzae*, *Listeria monocytogenes*, *Neisseria meningitidis*, *Streptococcus agalactiae*, and *Streptococcus pneumoniae*), seven viruses (*Cytomegalovirus* (CMV), *Enterovirus* (EV), *Herpes simplex virus* 1 (HSV-1), *Herpes simplex virus* 2 (HSV-2), *Human herpesvirus* 6 (HHV-6), *Human parechovirus* (HPeV), and *Varicella zoster virus* (VZV)), and one yeast (*Cryptococcus* (*C. neoformans*/*C. gattii*)).

## 2. Case Description

In January 2024, an 8-month-old patient arrived at a local hospital with parents reporting fever and diarrhea from the previous day. The same day, he experienced convulsive seizures. Hematochemical examinations were performed, revealing significant elevation in inflammation indices; hyponatremia was also found. Empirical antibiotic therapy with ceftriaxone was initiated. A head Computerized Axial Tomography (CT) scan was performed, which reported normal results. When transferred to our emergency department, the patient was in severe general condition. A lumbar puncture was immediately performed, and a cerebrospinal fluid (CSF) sample was collected. A follow-up head CT scan, prompted by clinical worsening, revealed areas of increased density with hemorrhagic regions at the bilateral temporoparietal level. Electroencephalogram tracing showed globally hypovolted and monomorphic brain activity and absent responsiveness to nociceptive stimuli. Thus, orotracheal intubation was performed and the patient was admitted to the Intensive Care Unit for appropriate treatment and further investigation.

## 3. Microbiological Investigation and Relevant Findings

**Figure 1:**
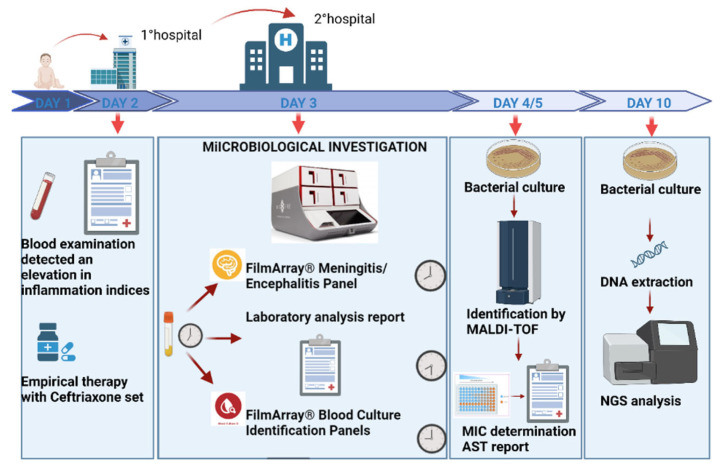
The timeline of microbiological investigations and relevant findings, starting from the third day of patient hospitalization. Created in BioRender [9]. Abbreviations: AST—antimicrobial susceptibility test.

On the same day as the patient’s arrival at our hospital, two CSF samples and one set of blood cultures were sent to the laboratories for chemical–physical analysis and molecular analysis to detect any microorganisms responsible for the infection. The CSF sample was immediately processed using the FilmArray Meningitis/Encephalitis Panel BioFire (bioMérieux), a molecular method capable of identifying the 14 most important pathogens involved in meningitis etiology (including only the K1 strain for *E. coli*, as previously mentioned) in approximately one hour. However, the test gave a negative result. At the same time, the report of the physical–chemical analysis of the same sample showed a cloudy appearance, lactescent color, and an increase in total protein (186.0 mg/dL). The white blood cell count was also elevated (>30 mm^3^, with a normal range of 0–5), together with the CSF lactate levels (>8.42 mmol/L, with a normal range of 1.10–2.80) and CSF albumin levels (>94.1 mg/dL, with a normal range of 14.00–35.00). Meanwhile, glucose levels were markedly depleted (<2 mg/dL, with a normal range of 60–80). The clinical manifestations and the results of the physical–chemical analysis were fully compatible with bacterial infection. A few hours later on the same day, after consultation with infectious disease specialists, we decided to use the FilmArray Blood Culture Identification (BCID) (bioMérieux) panel off-label. The BCID syndromic panel, which is not certified for this type of material, can identify 43 targets, i.e., 15 g-negative bacteria (including the generic target for *E. coli*), 11 g-positive bacteria, 7 fungi, and 10 antibiotic resistance markers, in about one hour. The BCID panel successfully identified the presence of a non-K1 *Escherichia coli* strain carrying the CTX-M resistance gene, an extended-spectrum beta-lactamase (ESBL). The CSF sample positive on the BCID FilmArray was immediately seeded on agarized culture plates to isolate the microorganism. The strain grown on the plates was successfully identified by matrix-assisted laser desorption/ionization–time-of-flight mass spectrometry (MALDI-TOF), which confirmed *E. coli* identification; then, a phenotypic antibiogram was set up to determine antibiotic sensitivity. The next day, the blood culture set turned positive, confirming the presence of an *E. coli* with the same CTX-M-type resistance mechanism. The *E. coli* strain was also characterized by whole-genome sequencing (WGS) after DNA extraction, in order to study the antimicrobial resistance (AMR) and virulence factors of the strain (Figure 1). WGS analysis confirmed the presence of an *E. coli* ST131 strain, serotype O25:H4 *fumC*40/*fim*H30, belonging to phylotype B2 (TspE4+, arpA−, chu+, yjaA+), a resistant high-risk clone often associated with sepsis [5] and urinary tract infections [6]. Regarding AMR, the strain carried several genes, including those encoding the ESBLs CTX-M-15 and TEM-1B, present on a single plasmid together with other resistance genes, as well as *gyrA* and *parC* mutations associated with fluoroquinolone resistance (see Supplementary Tables S1 and S2). In terms of virulence, more than 150 genes were identified, coding for factors involved in several mechanisms (see Supplementary Table S1). Among the plasmids predicted from the assembled genome, one was found to be similar (82.9% query coverage and 98.5% identity) to *E. coli* pS286colV (GenBank acc. n° HF922624), carrying a Conserved Virulence Plasmidic Region (CVP), which had initially been found in extra-intestinal avian pathogenic *E. coli* (APEC) [7] and linked to meningitis cases caused by non-K1 *E. coli* [8] (see Supplementary Figure S1 and Table S2).

## 4. Discussion

Currently, the presence of the K1 capsule target is essential for the rapid recognition of *E. coli* responsible for meningitis using the available multiplex molecular tests. However, as in the case reported here, microorganisms not included in a specific syndromic panel can also cause meningitis. Indeed, the syndromic meningitis panel did not allow the diagnosis of the non-K1 *E. coli* strain carrying CTX-M, due to the absence of the appropriate targets. Instead, the off-label use of the syndromic BCID panel allowed us to identify the presence of an *E. coli* and its resistance mechanism at an early stage in a case that would otherwise have been reported as negative. In our case, unfortunately, the child died 5 days after the onset of symptoms, despite our ability to identify the pathogen and its resistance mechanism on the same day as his arrival at our hospital. He had arrived in our center on the third day in very critical condition, already on empirical cephalosporin therapy set by the previous hospital, which later proved ineffective given that the strain was an ESBL-producing *E. coli*. The patient was already severely compromised despite the therapeutic shift to carbapenem, specifically meropenem at a dose of 40 mg/kg every 8 h, which was made just a few hours after his arrival, following the result of the syndromic BCID panel. Given the peculiar virulence of the bacterial isolate, we decided to perform a sequencing analysis to better understand and characterize this *E. coli* strain, despite the patient already being deceased. Based on the WGS results, we could conclude that the increased virulence of this high-risk clone, combined with its resistance mechanisms, likely played a key role in the progression to a fatal outcome. This case also demonstrated that the use of off-label panels with a larger number of targets, at the right time and in the right situation, could allow the result of the culture examination to be anticipated within approximately 24–48 h for identification and 48–72 h for phenotypic antibiogram results. Moreover, the use of state-of-the-art technologies such as WGS allowed us to better characterize resistance mechanisms and their mobilization and to study virulence determinants that would otherwise be impossible to assess with current techniques used in clinical practice.

## 5. Methods

### 5.1. Matrix-Assisted Desorption/Ionization–Time-of-Flight Mass Spectrometry (MALDI-TOF MS)

The *E. coli* isolate was cultured on Columbia agar plates with 5% sheep blood (bioMérieux, Marcy-l’Étoile, France) and incubated overnight at 37 °C. The next day, the colonies grown on plates were typed by matrix-assisted laser desorption/ionization–time-of-flight mass spectrometry (MALDI-TOF MS; Bruker Daltonics, Bremen, Germany). The *E. coli* strain was tested for antimicrobial susceptibility using the MicroScan WalkAway system (Beckman Coulter Inc., Brea, CA, USA) and by interpreting the results according to clinical breakpoints given by the European Committee on Antimicrobial Susceptibility Testing (EUCAST) tables (version 14.0 [10]).

### 5.2. Genomic DNA Extraction and Whole-Genome Sequencing (WGS)

The extraction of bacterial DNA was carried out with the automatic extractor EZ1 (Qiagen BioRobot EZ1) using the extraction kit (EZ1&2 DNA tissue kit, Qiagen, Hilden, Germany), with the elution volume set at 50 µL, following the manufacturer’s instructions. The extracted DNA was quantified using a Bioanalyzer Instrument, and the libraries for Next-Generation Sequencing were generated using the DNA Library Prep kit (Illumina, San Diego, CA, USA) and sequenced on an Illumina MiSeq instrument (Illumina, San Diego, CA, USA) using the MiSeq Reagent Kit v3 to obtain 2 × 250 bp paired-end reads.

### 5.3. Bioinformatic Analysis

The obtained raw reads were pre-processed by trimming for adapters and quality (with Phred score > 28) using Fastp (v0.23.4 [11,12]). After trimming, a total of 1.8 million reads were obtained, which were quality-checked with FastQC (v0.11.9 [13]). To determine the taxonomic classification and screen for potential contaminations, Kraken2 (v2.1.3 [14]) was used. Reads were then assembled *de novo* by using SPAdes (v3.15.5 [15]), and the quality of the obtained assemblies was evaluated using Quast (v5.1 [16]). Bacterial annotation was performed using Prokka (v1.14.6 [17]). To determine the strain characteristics, Multilocus Sequence Typing (MLST) was performed with the mlst tool (v2.11 [18]), subtyping based on the *fumC* and *fimH* alleles was performed with CHTyper (v1.0 [19]), and the silico EzClermont tool (v0.6.3 [20]) was used to infer the phylotype. Antimicrobial resistance (AMR) genes and virulence factors were searched using ABRicate (v0.4 [21]) with the Comprehensive Antibiotic Resistance Database (CARD [22]) and the Virulence Factor Database (VFDB [23]), respectively. Mobile genetic element (MGE) identification and plasmid reconstruction were carried out using the MOB-suite tool (v3.1.8 [24]). Raw reads were then mapped using bwa mem [25] against plasmid sequences identified with MOB-suite to determine query coverage and percentage identity.

To investigate fluoroquinolone resistance, mutations in *gyrA* (encoding the subunit A of DNA gyrase—primary FQ target) and *parC* (subunit A of topoisomerase—secondary FQ target) were analyzed and compared to the sequences of *Escherichia coli* str. K-12 substr. MG1655 (GenBank acc number NC_000913).

Single-nucleotide polymorphism (SNP) calling was performed with Snippy (v4.6.0 [26]), using the ST131 reference genome *E. coli* NCTC13441 (GenBank accession number NZ_UFZF01000006.1) as a reference, removing regions predicted to be possible recombinogenic regions by Gubbins (v3.3.1 [27]). Reference strains for several ST131 *E. coli* strains available from the PATRIC database [28] and from the NCBI database [29] were also incorporated. The core SNPs identified relative to *E. coli* NCTC13441 were used to infer phylogenetic relationships among the whole-genome sequences by maximum likelihood (ML) using IQTREE (v2.0.6 [30]), with 1000 bootstrap replicates under the best nucleotide substitution model (TVM + F + ASC + R5) determined by ModelFinder [31]. The ML tree was visualized and annotated using iTOL (v6.5.2 [32]).

## 6. Conclusions

In the era of fast microbiology, the genomic characterization of emerging non-K1 or K-negative *E. coli* strains can help to shed light on these new pathogens. This may suggest their inclusion in new multiplex PCR assays, independent of capsular genes, for more accurate and rapid meningitis diagnosis [33]. In addition, this case underscores the importance of integrating different approaches in the diagnosis of bacterial meningitis, in which mere microbiological diagnosis based upon validated methods should be integrated with other tests (perhaps not validated for the specific matrix, like CSF in this case), if clinical evidence suggests and/or requires it. Considering our clinical experience, where molecular tests are increasingly effective but (currently) also much more specific than culture methods, we propose a workflow involving both infectious disease specialists and microbiologists to synergistically interpret test results and guide therapeutic decisions. This would allow us to not rely just on specific bacterial targets, which might not always be present, for the rapid identification of pathogens.

## Data Availability

The sequence data obtained in this study are openly available on the European Nucleotide Archive (ENA) under accession n. PRJEB82691.

## Supplementary Materials

The following supporting information can be downloaded at https://www.mdpi.com/article/10.3390/antibiotics13121144/s1, Supplementary Materials: Figure S1: Maximum likelihood phylogenetic tree clinical isolate and 78 reference genomes belonging to ST131; Table S1: Antimicrobial resistance and virulence genes identified; Table S2: Plasmids identified in the samples.
