# Supplementary material for: Integrating Diagnostic Approaches in Infant Bacterial Meningitis Caused by a Non-K1 Escherichia coli: A Case Report
Source: Antibiotics (Basel). 2024 Nov 28;13(12):1144. doi: 10.3390/antibiotics13121144 (PMC11672694; doi:10.3390/antibiotics13121144)
Supplement: Supplementary file 1 [file antibiotics-13-01144-s001.zip › antibiotics-3266459-supplementary.pdf]

Table S1. Antimicrobial resistance and virulence genes identified.

Gene	Coverage (%)	Identity (%)	Accession number
**Antimicrobial Resistance genes**			
mdfA	100.00	96.27	JQ394987:0-1233
kdpE	99.26	95.54	U00096.3:721733-721055
acrA	100.00	99.08	U00096.3:485619-484425
acrB	100.00	98.76	U00096.3:484403-481253
ampH	100.00	97.06	AP012030.1:396711-395553
H-NS	100.00	99.28	BA000007.3:1738104-1737690
mdtH	100.00	98.35	U00096:1125326-1124117
mdtG	100.00	98.37	CP000800.1:1192954-1191727
msbA	100.00	98.06	U00096.3:966620-968369
evgS	100.00	96.33	U00096:2484373-2487967
evgA	100.00	99.02	BA000007.3:3212025-3212640
emrK	100.00	97.73	D78168:536-1592
emrY	100.00	97.66	D78168:1591-3130
pmrF	100.00	97.63	U00096:2367070-2368039
yojI	100.00	97.38	U00096.3:2308615-2306971
baeR	99.86	97.09	AP009048.1:2166412-2167135
baeS	100.00	90.53	AP009048:2165012-2166416
mdtC	100.00	93.99	U00096:2158385-2161463
mdtB	100.00	96.16	U00096:2155262-2158385
mdtA	100.00	95.59	U00096:2154015-2155263
ugd	100.00	95.97	U00096:2099613-2098446
mdtM	100.00	95.05	U00096.3:4568519-4567286
CRP	100.00	99.21	AP009048.1:4154296-4153663
mdtE	100.00	98.53	AP009048.1:3981183-3980025
mdtF	100.00	97.30	U00096:3660413-3663527
gadW	100.00	100.00	CP015085.1:2552440-2551711
gadX	100.00	93.94	AP009048.1:3974604-3975429
tolC	100.00	98.19	FJ768952:0-1488
bacA	99.76	98.29	U00096.3:3204131-3203309
acrD	100.00	98.65	AP009048.1:2586250-2589364
ampC	100.00	97.80	U00096.3:4378944-4377810
mdtP	100.00	97.61	AP009048.1:4304509-4303042
mdtO	100.00	97.08	AP009048.1:4306557-4304505
mdtN	100.00	96.22	AP009048.1:4307588-4306556
eptA	100.00	91.42	AP009048:4340268-4338624
marA	100.00	98.96	AP009048.1:1621287-1621671
acrS	100.00	98.34	U00096:3413465-3412802
acrE	100.00	98.79	U00096:3413863-3415021
acrF	100.00	96.59	U00096:3415032-3418137
cpxA	99.56	98.54	BA000007.3:4905062-4903688
emrE	100.00	92.19	Z11877.1:485-818
emrR	100.00	98.49	U00096.3:2810769-2811300
emrA	100.00	98.04	AP009048:2810082-2811255
emrB	100.00	98.44	U00096:2812615-2814154
aadA5	100.00	100.00	AF137361:63-852
dfrA17	100.00	100.00	DQ838665:0-474
tet(B)	100.00	99.75	AB089595:0-1206
OXA-1	100.00	100.00	JN420336.1:2230-1399
AAC(6')-Ib-cr	100.00	100.00	DQ303918:0-600
APH(6)-Id	100.00	99.88	AF024602:3155-3992
APH(3'')-Ib	100.00	99.75	AF313472:15593-16397
sul2	100.00	99.63	AY055428.1:21084-20268

TEM-1	100.00	99.88	AL513383:161910-162771
CTX-M-15	92.47	100.00	AY044436:1435-2311
**Virulence genes**			
aslA	100.00	98.18	AAG10151
cheB	97.52	75.15	YP_001006775
cheW	90.36	74.89	YP_001006779
cheY	99.49	77.06	YP_001006774
cheZ	87.91	70.71	YP_001006773
chuA	100.00	99.54	NP_756170
chuS	100.00	99.03	NP_756169
chuT	100.00	99.80	NP_756175
chuU	100.00	99.50	NP_756179
chuV	100.00	99.75	NP_756180
chuW	100.00	98.80	NP_756176
chuX	100.00	100.00	NP_756177
chuY	100.00	98.24	NP_756178
csgA	99.78	73.48	NP_460115
csgB	99.78	83.81	NP_460114
csgC	97.25	74.38	NP_460116
csgD	100.00	81.57	NP_460113
csgE	98.48	79.29	NP_460112
csgF	99.04	80.76	NP_460111
csgG	100.00	83.21	NP_460110
cvaC	100.00	98.13	NP_752614
entA	100.00	99.42	NP_752613
entB	100.00	98.74	NP_752611
entC	85.86	96.68	NP_752599
entD	100.00	98.88	NP_752612
entE	100.00	98.20	NP_752604
entF	100.00	97.44	NP_752609
entS	99.93	97.41	YP_002390132
etsA	100.00	98.93	NP_752600
fdeC	100.00	98.43	NP_752610
fepA	100.00	99.14	NP_752606
fepB	100.00	97.84	NP_752608
fepC	100.00	97.89	NP_752607
fepD	100.00	99.00	NP_752602
fepG	99.83	90.76	NP_757241
fes	100.00	98.90	NP_757243
fimA	100.00	98.52	NP_757244
fimC	100.00	98.83	NP_757240
fimD	100.00	96.44	NP_757245
fimE	100.00	98.21	NP_757247
fimF	100.00	97.48	NP_757248
fimG	100.00	98.52	NP_757242
fimH	97.53	72.80	YP_001006763
fimI	80.03	74.25	YP_001006762
flgC	98.93	75.66	YP_001006759
flgD	80.70	79.35	YP_001006758
flgG	96.19	71.66	YP_001006757
flgH	99.13	75.08	YP_001006770
flgI	95.19	75.22	YP_001006783
flhA	94.47	73.75	YP_001006726
flhC	99.50	78.75	YP_001006742
fliA	92.57	76.20	YP_001006744
fliG	99.50	76.43	YP_001006748

fliI	98.40	77.29	YP_001006751
fliM	92.96	72.76	YP_001006752
fliP	100.00	99.80	NP_405467
fliQ	75.56	71.85	NP_439337
fyuA	100.00	93.86	YP_404599
gmhA/lpcA	100.00	96.06	YP_404600
gspC	100.00	94.98	YP_404601
gspD	100.00	94.25	YP_404602
gspE	100.00	96.27	YP_404603
gspF	100.00	94.73	YP_404604
gspG	100.00	92.20	YP_404605
gspH	100.00	94.39	YP_404606
gspI	100.00	96.52	YP_404607
gspJ	100.00	94.08	YP_404608
gspK	99.05	93.08	YP_404609
gspL	98.79	71.00	YP_094724
gspM	87.41	70.83	NP_933683
hlyF	99.91	98.37	NP_753168
htpB	100.00	99.14	NP_753167
IlpA	100.00	98.62	NP_753166
iroB	100.00	99.48	NP_753165
iroC	100.00	99.13	NP_753164
iroD	100.00	99.76	NP_405471
iroE	100.00	99.46	NP_405472
iroN	100.00	99.83	NP_709454
irp1	100.00	99.79	NP_709455
irp2	100.00	98.16	NP_755500
iss	98.80	96.30	NP_755499
iucA	99.96	88.41	NP_755498
iucB	98.60	71.14	NP_439706
iucC	100.00	98.33	AAA21682
iucD	100.00	95.37	AAA24046
iutA	87.42	73.77	AAA24047
kdsA	82.66	70.63	NP_230208
kpsD	98.42	72.44	YP_001006782
kpsM	100.00	95.82	AAF37887
kpsT	100.00	99.36	
luxS	100.00	99.88	NP_755465
mchE	100.00	100.00	NP_755464
motA	99.44	77.60	NP_755460
ompA	100.00	99.25	NP_755459
ompT	100.00	99.11	NP_755458
papB	91.67	97.40	NP_755466
papC	100.00	94.02	NP_755468
papD	100.00	94.87	NP_755468
papE	100.00	100.00	NP_755463
papF	100.00	100.00	NP_755461
papG	100.00	100.00	NP_755457
papH	100.00	97.64	NP_755457
papI	98.60	70.27	NP_439271
papJ	99.74	98.01	NP_286006
papK	100.00	99.21	NP_286007
papX	100.00	98.06	NP_286009
rfaD	100.00	98.47	NP_286010
sinH	100.00	99.27	NP_405473
yagV/ecpE	100.00	99.81	NP_405468

yagW/ecpD	100.00	99.61	NP_405474
yagX/ecpC	100.00	99.56	NP_405475
yagY/ecpB	100.00	99.85	NP_405477
yagZ/ecpA	100.00	99.63	NP_405469
ybtA	100.00	99.73	NP_405470
ybtE	100.00	99.69	NP_405476
ybtP	100.00	99.49	NP_286011
ybtQ	100.00	100.00	CCV19846.1
ybtS	100.00	100.00	CCV19855.1
ybtT	100.00	100.00	NZ_CP083638.1
ybtU	100.00	100.00	CCV19883.1
ybtX	100.00	100.00	CCV19853.1
ykgK/ecpR	100.00	100.00	CCV19885.1

Table S2. Plasmids identified in the samples. The results given by MOB-suite tool are reported: primary cluster ID, replicon type, relaxase type, the accession number of the mash nearest neighbor. The presence of antimicrobial resistance genes (AMR) and virulence factors (VF) is also reported.

Plasmid	Primary cluster ID	Replicon type	Relaxase type	Mash nearest neighbor	AMR	VF
plasmid1	AA176	IncFIB	MOBP	HF922624		*iroB,iroC,iroD,iroE,iroN, ompT, iss, hlyF, mchF, etsC, cvaC*
plasmid2	AA337	IncFIA,IncFIC/ IncFII(29) (MGE)	MOBF	CP041523		*traJ, traT*
plasmid3	AA521	Col440II (MGE)	MOBP,MOBP	CP036198		
plasmid4	AA977	Col156	MOBQ	CP040268		
plasmid5	AC082	IncQ1		NC_019091	*aadA5, drfA17, tet(B) , APH(6)-Id, APH(3'')-Ib, sul2 , TEM-1B , CTX-M-15*	
plasmid6	AD486	Col440I		CP019023		

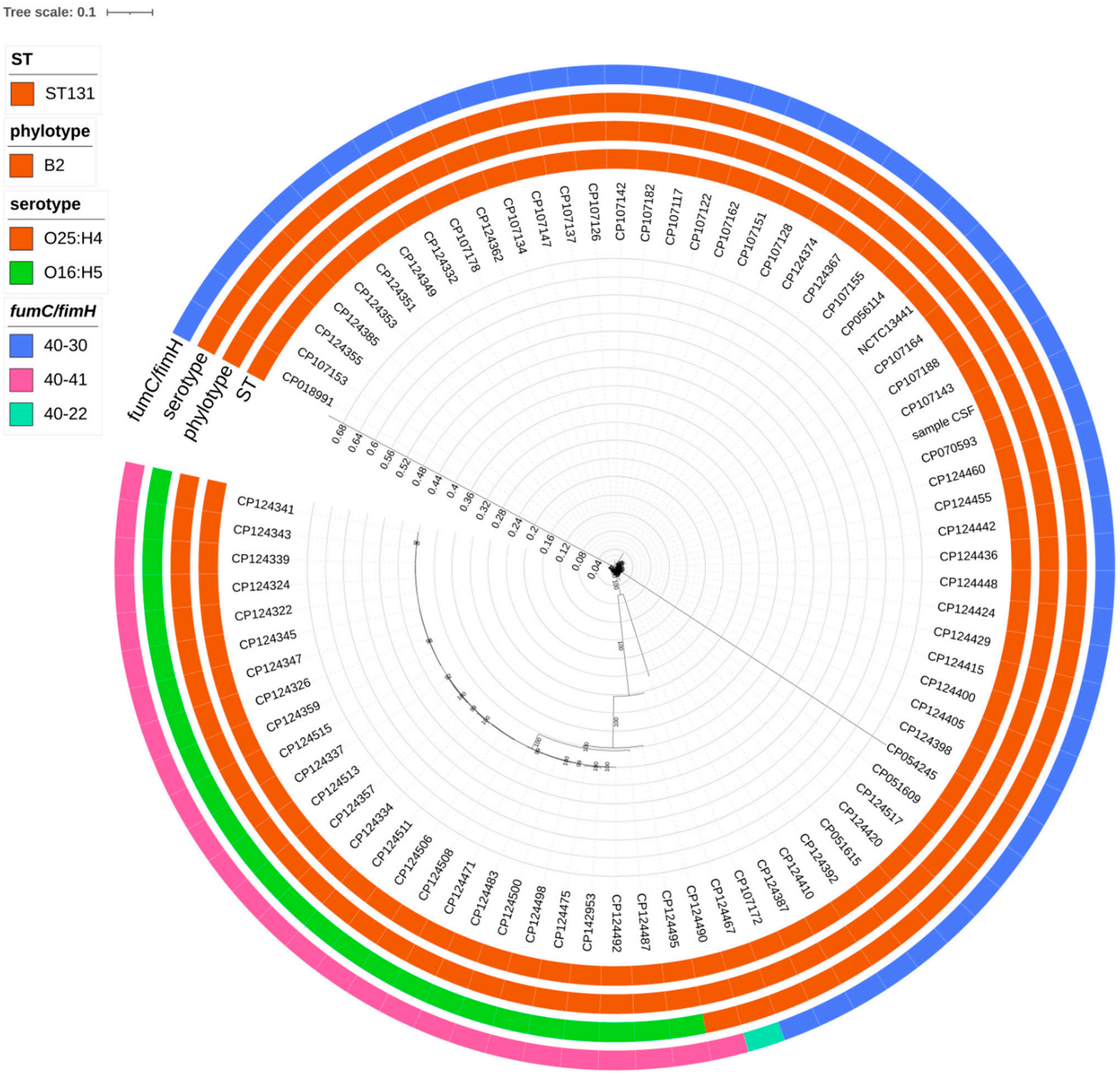

Figure S1: Maximum Likelihood phylogenetic tree of the clinical isolate and 78 reference genomes belonging to ST131. The phylogeny was estimated on a coreSNP of 30,577 bp with IqTree, using the best-fit model of nucleotide substitution TVM+F with 1,000 replicates fast boostrapping. Leaves number represent sample IDs, and bootstrap values higher than 90 are reported on branches. Information regardin the samples are reported: Sequence Type (ST=, phylotype, serotype and subtype based on *fumC/fimH* alleles.
